# Interventional nanotheranostics in hepatocellular carcinoma

**DOI:** 10.7150/ntno.80120

**Published:** 2023-01-09

**Authors:** Vivek P. Chavda, Pankti C. Balar, Srushti B. Patel

**Affiliations:** 1 Department of Pharmaceutics and Pharmaceutical Technology, L.M. College of Pharmacy, Ahmedabad, India.; 2 Pharmacy Section, L.M. College of Pharmacy, Ahmedabad, India.; 3 Pharmacy Section, Government Pharmacy College, Gandhinagar, India.

**Keywords:** Interventional Nanotheranostics, HCC, Nanoparticle, Liver Cancer, SELEX

## Abstract

Interventional nanotheranostics is a system of drug delivery that does a dual function; along with the therapeutic action, it also does have diagnostic features. This method helps in early detection, targeted delivery, and the least chances of damage to surrounding tissue. It ensures the highest efficiency for the management of the disease. Imaging is the near future for the quickest and most accurate detection of disease. After combing both effective measures, it ensures the most meticulous drug delivery system. Nanoparticles such as Gold NPs, Carbon NPs, Silicon NPS, etc. The article emphasizes on effect of this delivery system in the treatment of Hepatocellular Carcinoma. It is one of the widely spreading diseases and theranostics is trying to make the scenario better. The review suggests the pitfall of the current system and how theranostics can help. It describes the mechanism used to generate its effect and believes that interventional nanotheranostics do have a future with rainbow color. The article also describes the current hindrance to the flourishing of this miraculous technology.

## Introduction

Hepatocellular carcinoma (HCC) is continual primary liver cancer. It is the concern of a vast population. It is the fourth most common cause of cancer mortality and the sixth most frequent carcinoma overall 1. The main culprit behind being catastrophic is the lack of diagnosis at the early stage due to the absence of symptoms. Approximately, 90% of all primary hepatic malignancies are hepatocellular carcinomas, which pose a serious global health issue 2. The World Health Organization predicts that more than 1 million people would pass away from liver cancer in 2030, based on yearly predictions 3. Despite being a worldwide issue, the prevalence of HCC is not evenly distributed. Eastern Asia and sub-Saharan Africa have the highest rates of HCC. Additionally, it poses a significant concern in countries like the United States, Europe, and Japan 4. The topographical risk factor is always shifting due to vaccination rates and current advancements in medical treatments. As discussed in the staging system, there are multiple bodies with different biases of classification. Although not completely reliable, Barcelona Clinic Liver Cancer (BCLC) is the most accepted staging system to date. Very early stage (0) has a single nodule and early stage (A) have single or up to three nodule formation. Till the intermediate stage (B), which has multifocal tumor formation limited to the liver, all stages are asymptomatic as liver function is preserved. In the advanced stage (C), extrahepatic spread along with portal invasion is observed. Patients with end-stage illness (D) have severe cancer-related symptoms or have impaired liver function and here even liver transplantation cannot improve the condition 5.

## Occurrence

The occurrence of HCC is a tedious and complex process. The basic pathology of HCC is hepatocyte necrosis and regeneration, together with fibrotic deposition, which are all factors in the multistep, complicated process that leads to the development of HCC. The risk appears after cirrhosis is developed, and it rises along with deteriorating liver function 6. It is affected by various factors and yet we are not able to find the major reason for the HCC. However, we have some influencing factors to accelerate or worsen the condition. Predominantly, the viral load of diseases such as hepatitis B and hepatitis C 7. Along with this, other high-risk factors include mutation of genes, non-alcoholic fatty liver disease (NAFLD), aflatoxin, metabolic disorders such as diabetes, obesity, and many more.

Hepatitis B, having partial double-stranded circular DNA, leads to chronic hepatitis at the initial stage. Cirrhosis develops as a result of the body's immunological response, which includes inflammation, oxidative stress, and the necrosis of liver cells. Telomerase is activated again by cirrhosis in cells. Telomeres shorten with each subsequent cell division, however, in malignant or defective cells, telomerase is reactivated, preventing it from happening 8. This resulted in atypical cell division and the development of HCC. It is observed that there are 100 folds more chances of a person with HBV compare to a normal individual 9. In addition to this consequence, it also caused chromosomal instability and the loss of a tumor-suppressing gene. When this gene is altered, the defective gene is not deleted, which might again result in HCC. Similarly, hepatitis C leads to successive cirrhosis and at last HCC. HCC still has a high rate of morbidity and death due to the rising of cirrhosis in HCV. Both viral diseases are the major cause of HCC 10. The most frequent somatic change in HCC is aberrant telomerase reverse transcriptase (TERT) activation, which can occur as a result of promoter mutations, viral integrations, or localized amplification 11.

Aflatoxin B1 is frequently ingested together with contaminated grains, and liver cytochrome p450 breaks it down. Once digested, it functions as a DNA mutator to cause the transversion of G to T in TP53's codon 249's third position 12. HBV and aflatoxin show a synergistic action. NAFLD progression leads to inflammatory response and hence the risk factor for the development of HCC. Diabetes is a contributing factor and it is observed that a person with a high body-mass index and insulin resistance is more susceptible 13. Talking about environmental factors is crucial as they influence the progression of the disease. Excessive alcohol consumption leads to the destruction of liver cells and causes inflammation and necrosis. It intensifies the symptoms and progression of HCC 14. Tobacco, consumed when a person is already infected by viral disease (HBV/HCV) exaggerated the situation.

## Complications

HCC is a dangerous condition that necessitates prompt diagnosis and treatment to prevent further deterioration. It enables the doctor to treat the patient's symptoms and enhance both the patient's life expectancy and lifestyle. The accurate detection helps to find the core error in the body, along with its immune system to concentrate the treatment. Facing problems in treatment for world's one of the most prevalent diseases is a burden on the health care system that needs to resolve.

### In Diagnosis

The progression of HCC completely relies on the stage when the disease is successfully diagnosed. Patients which are diagnosed with cirrhosis often develop HCC. It can cause hepatic problems such as hepatic encephalopathy, portal vein thrombosis, worsening ascites, variceal hemorrhage, obstructive jaundice, and pyogenic liver abscess. A potentially fatal HCC consequence is intraperitoneal hemorrhage 15 16. There are different types of diagnostic methods to confirm HCC such as X-Ray, Magnetic Resonance Imaging (MRI), Computed Tomography (CT)scam, Liver Biopsy, Serum Alpha-Fetoprotein (AFP), Ultrasonography, and many more. Among these, all diagnostic tests, liver ultrasonography, and serum alpha-fetoprotein are the two most popular methods. For non-invasive techniques, liver ultrasonography and serum AFP are the most popular ones whereas liver biopsy is preferred as the invasive method 17.

Liver ultrasonography is an imaging technique that uses highly intense sound waves which gives real-time images or videos of internal body structure like organs, soft tissues, and blood vessels. It also gives information regarding the stage of HCC but the detection of the type of focal liver lesion (FLL) accurately and the recognition between HCC and non-HCC, particularly in cirrhosis and other chronic hepatic condition presence is the major challenge in conventional liver sonography 18. Another complication is associated with instrument standardization and patient disease condition as it varies from person to person. Thus, regular screening is required to avoid minor challenges 19.

AFP is another common diagnostic method after liver ultrasonography for the detection of HCC. AFP is an important tumor biomarker as a diagnostic tool. The level of AFP elevates in three conditions: liver cancer or testicular cancer, pregnancy, and sometimes in non-cancerous diseases such as cirrhosis and hepatitis. It can lead to a false assumption about HCC. Serum AFP levels in healthy persons normally range from 5 to 10 ng/mL. However, the AFP level of 400 ng/ml is a set as prejudicial between hepatocellular cancer and chronic diseases such as cirrhosis or hepatitis. Due to this reason (less sensitivity and accuracy) some controversial studies have a disagreement with the usage of AFP level as a screening method in HCC patients 20. The AFP measurement is unsatisfactory since 80% of cases of small hepatocellular carcinoma do not show an elevation in AFP values which is one of the limitations of this diagnostic method 21.

Liver biopsy, being an invasive method, has drawbacks due to patient discomfort. NASH must be definitively diagnosed, and liver biopsy is regarded as highly helpful in separating NASH from other disorders, determining a prognosis, and evaluating the outcomes of therapeutic intervention. However, liver biopsy has several limitations and is often ineffective in non-advanced patients. Furthermore, even if NASH is diagnosed by liver biopsy, there is no known therapy for it. Pathologists disagree in their diagnosis and recognition of liver biopsy results. Additionally, there is a chance of “tumor seeding” during a biopsy, in which case cancer cells spread to other tissues along the path the biopsy instrument takes. For individuals with the usual imaging characteristics of HCC, liver biopsy is not commonly performed due to these hazards 22. During a liver biopsy, only 1/50000 of the entire liver tissue is collected, raising questions about sampling inaccuracy 23.

### In Treatment

Treatments for HCC are complicated because enormous factors influence the progression of the disease. Methods like liver transplantation, ablative techniques like radiofrequency ablation, and many more are used.

Liver transplantation leads to the removal of the cancerous part of the liver and the introduction of the healthy liver part from a donor 24. The major drawback of this system is high demand compare to less availability of donors. Also, there is the possibility of rejection due to the action of the immune system 25.

Concerning the molecular level, angiogenesis is a major factor influencing the growth of tumors 26. There is a system to reduce this phenomenon to block growth. Drugs like Sorafenib acts by blocking the VEGF-R pathway, highlighting the promise of antiangiogenic treatments for HCC 27. For a better therapeutic result, it is necessary to investigate the redundant and complicated angiogenic pathways. Despite being accepted as a life-extending medication for individuals with advanced HCC, sorafenib's effectiveness is highly reliant on the causes of HCC and hence is not completely reliable. Metastasis is also a serious problem of the tumor cells which hurdles the conventional system of treatments 28.

### Need for a novel system

Considering all the shortcomings of diagnosis and therapy, it becomes clear that another approach must be found immediately. We cannot rely on a single technique to identify HCC during the diagnostic phase. To guarantee the outcomes, regular testing is required. Additionally, using the conventional way of diagnosis makes early-stage detection exceedingly challenging. Additionally, some accurate tests are intrusive, which is not what patients or doctors desire. In terms of treatment, a variety of medications and approaches are necessary to produce the desired pharmacological effect 29. Additionally, even a well-known procedure like liver transplantation is ineffective for treating end-stage HCC 30. Healthcare systems require a medication delivery system that can improvise both diagnostics and therapy with the least amount of intrusion and most impact. For that reason, The prodigious concept of Nanotheranostics same in the lime light 31.

## Introduction to Nanotheranostics

Nanotheranostics is a rapidly expanding field in medicine, that combines diagnosis and therapeutics by using nanoparticles (NPs). In recent years, Nanotheranostics platforms have been suggested as a promising field in the management of cancers. The difficulties of traditional chemotherapy have been steadily getting easier because of developments in nanotechnology-based medicines delivery methods. Due to its increased efficacy and decreased side effects, nanotechnology contributes to the effective delivery of drugs to damaged tissues. Lipidic and polymeric composite nanomedicines offer a better delivery system and create novel therapeutic approaches for cancer like HCC 32,33. Late diagnosis, the complexity of treatment, reoccurrence, and multidrug resistance lead to an increase in the mortality rate of HCC. In addition, most of the cases are diagnosed in the advanced stage of HCC so, there is a need to detect it in the early stage and ace the burden of treatment, to overcome this hurdle NPs are used as a diagnostic tool as well as a therapeutic agent nowadays [34, p. 1]. The overall potential of nanotheranostics agents is greater than traditional ones owing to simultaneous diagnosis and site-specific therapy due to which they are widely applicable 35,36. They have unique benefits like biocompatibility, decreased toxic effects, good stability, improved permeability, more retention in tumor cells, and specific targeting ability, nanoparticles size ranges from about 1 to 1000 nm, and have the potential to treat cancer 37. Moreover, NPs may shield the enclosed compounds from intracellular and extracellular deterioration, enhancing intracellular bioavailability but it is also affected by NPs' size, shape, functional group, etc. Different types and different shapes of NPs are also available and used according to disease pathogenesis.

**Figure 1:**
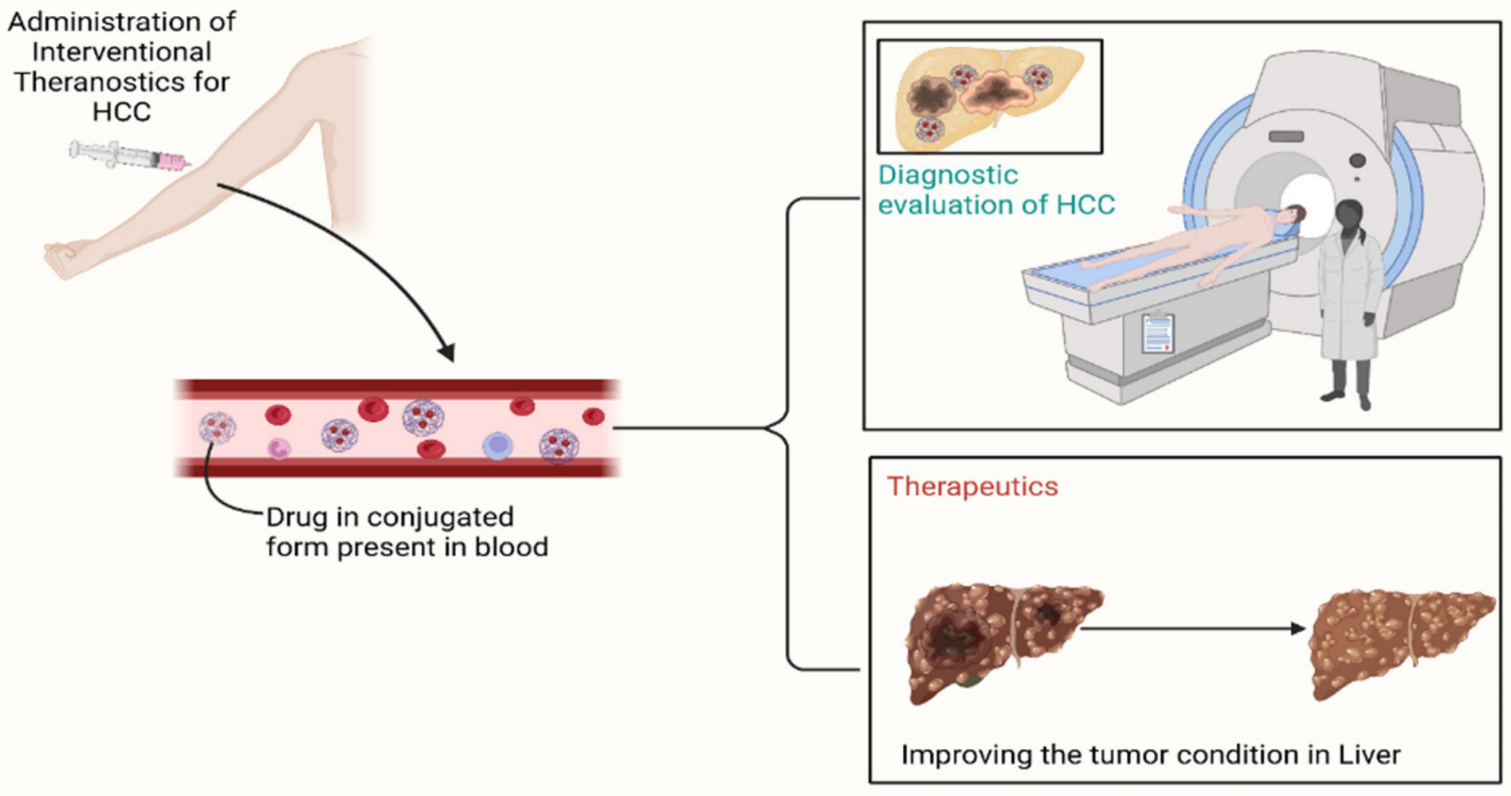
** Illustration of the overall working of the interventional drug.** The molecule, due to binding with particles such as nanoparticles, has gained its site-specific property. Due to it, the delivery of drugs becomes way easier and free from systemic toxicity. Along with that, the attached molecule consists of special characteristics, which lead to the identification of the location of the tumor present in the body. To sum up, it holds both therapeutic and diagnostic properties simultaneously.

NPs are colloidal structures composed of various polymers and active drugs. There are different types of NPs such as organic NPs, inorganic NPs, and hybrid NPs. The research conducted on various inorganic NPs which are used in HCC includes gold NPs (GNPs/AuNPs), alumina NPs, arsenite NPs, calcium NPs, chitosan NPs, iron oxide NPs, polyethylene NPs, platinum NPs, polysaccharide NPs, selenium NPs, silica NPs, silver NPs, etc. these are used as nano vehicles to carry anticancer drug/cargo to the site of action. To achieve these NPs various gene engineering or genetic modification methods are used 31,32. Nanocarriers are regarded as having a high degree of site-specificity and therapeutic efficacy when it comes to the clinical delivery of a chemotherapeutic drug 32. The polymeric coating with different polymers can also affect NPs' therapeutic action, cellular uptake, stability, and drug release at the cancer site. Various biodegradable polymers such as polyethylene glycol (PEG), natural polymers like chitosan, alginate, and synthetic polymers like polyesters are commonly used in nanoparticle formulation. However, inorganic gold NPs (GNPs) get more recognition due to their less toxicity and biological inertness 38-40. NPs or nanomedicines should formulate in such a way that they should have enough bioavailability and be able to overcome the challenges of a conventional drug (Figure 1). NPs are suggested as they cumulate around the tumor site and release slowly which increased permeation or diffusion and retention time at the disease site 41,42.

Apart from the therapeutic application of NPs, they are also used to diagnose and detect the early stage of HCC with the help of nanotechnologies because of their electrical, optical, and magnetic properties 43. Nanomaterials identify specific biomarkers which assist to diagnose HCC. Biomarkers are specific and different in various diseases and also provide information about disease progression. hence, the measurement of biomarkers through NPs proves a powerful tool. These types of nanomaterial are also known as nanosensors. They are capable to measure a large number of biomarkers in small samples. NPs that are coupled to a specific ligand often form the basis of nanosensors. The ligand identifies the target molecule, while the nanoparticle functions as a signal generator or detector, and together they determine sensitivity, these make nanosensors highly specific. Various NPs such as metallic NPs, magnetic NPs, or nanotubes are used to detect targets like antibodies, proteins, enzymes, etc. 44,45. Drug development is a time-consuming, expensive endeavour that calls for multidisciplinary skills 46. For the screening of drugs, molecular docking is virtual platform which is used in early phase of drug discovery 47. There are more than 250 approved drugs which uses bio-informatics tools in discovery procedure 48.

## Mechanism of action of nanoparticles in Hepatocellular Carcinoma

The NPs used to treat HCC have different physical and chemical properties which reflect in their delivery and mechanism by which they act and it is also affected by some other factors. So, it is not completely possible to standardize any one mechanism of NPs in the case of HCC but here, we review the cellular uptake mechanism that is most commonly followed by cargo polysaccharide NPs. Besides this, some NPs also respond to external stimulation like ultrasound stimulation, heat, pH, light, etc., these types of NPs can release drugs in a controlled manner which can increase more precision and targeting of drug delivery 49,50.

Gold NPs, liposomes, and chitosan-based NPs are the most common examples that are often used in the treatment of HCC 51. These NPs experience cellular uptake mechanisms in cancerous cells of the liver. Gold NPs used to carry drugs or active constituents on their surface while liposomes are filled with payload in the core of nanoliposome. The payload of NPs is facilitated by endocytosis into HCC cells, also they can penetrate via alternative pathways such as plasma membrane penetration (passive penetration). Different endocytosis processes like phagocytosis, pinocytosis, micropinocytosis, clathrin-based and caveolae-mediated endocytosis are followed by different NPs. Nanomaterials are encased within the initial endocytic vesicles during endocytosis, preventing their direct entry into the cytoplasm. While nanomaterials that are ingested by membrane penetration are immediately moved into the cytoplasm, which can be the better option in case of targeted drug delivery, after reaching their targeted site they release the drug from NPs and shows the cytotoxic effect on HCC cells 52,53. The drugs which have less solubility and absorption are formulated in NPs for targeted drug delivery which after reaching the cancerous site and releases drugs into the microenvironment of cancer cells and stays for a longer period 49. some of the NPs like gold nanoparticles may follow an active targeting mechanism if they are functionalized. In active targeting surface of NPs has been modified by attaching different proteins, antibodies, enzymes, and other targeting agents which results in increased efficacy of NPs by interacting with particular molecules on HCC cells.

It is crucial to target cancer-affected vasculature when treating cancer so, in this condition the lipid-based NPs provide safe and effective drug delivery at the specific cancer site. Currently, FDA-approved lipid-based NPs named liposomes are greatly used to treat HCC in which anticancer drug is enclosed with a phospholipid bilayer, providing protection against physical barriers and increasing the efficacy of the drug 54-56. Cancer is a multistep complex process. It's an abnormal cell growth that is characterized by new irregular blood vessel formation or angiogenesis (tumor vasculature). These blood vessels are leaky, and porous and have different endothelial membranes than normal blood vessels. Liposomes take advantage EPR effect (enhanced permeation and retention effect) of the leaking neo vasculature and passively concentrate near inflamed malignant cells, the EPR effect is caused by lack of adequate lymphatic outflow and increased permeability of tumor blood vessels 57-60. After reaches to the site of the cancer lipid-based, NPs are undergone endocytosis by the cellular uptake mechanism and release the anticancer drug from nanoparticle formulation and inhibit tumor growth or result in cancerous cell death. Liposome efficacy may be affected by different polymeric materials. Nowadays, biodegradable polymers like PEGylated liposomes (PLS) are frequently explored as they improve hydrophobic drugs' solubility, half-life, and efficacy via the EPR effect 61.

Gold NPs are also well known in the target-specific treatment of HCC. The gold NPs show their effect by inhibiting the cytochrome P450(CYP) enzymes at a cellular and molecular level. Gold NPs are mostly used in the detection of HCC. The surface of gold NPs bears a negative charge due to which we can modify it with different drugs, genes, and other biomolecules. Further, they bind with a specific target through which we can detect it. Moreover, gold NPs can also reduce chemotherapy resistance if they are given in conjugation with other drugs. Apart from this oxidative stress gene expression is also altered by gold particles which suggests that they also have apoptosis and necrosis cell death activity which may produce a toxic effect on healthy cells of the liver 62, 62,63. Another example of NPs that are used in HCC and still under research is chitosan NPs. These are continuous drug-release systems. The experimental study on chitosan NPs suggests a mechanism of action that cargo-loaded NPs cause internal irradiation and consequently, lead to increased cell death by apoptosis and DNA destruction in cancer-affected liver cells 64.

## Advantages over traditional therapy

Interventional nanothernostics is the emerging platform that is required to overcome the drawbacks of the traditional system for the management of HCC. Nanoparticles with dominant sensitivity and specificity have been developed to address the problem of ailment diagnosis that occurs too late 65. This approach facilitates the targeted accumulation of therapeutic payload inside tumor lesions. They have the property of tissue penetration. This helps them to detect even the minute tumor in the liver. They selectively bind to the tumor cell and help in diagnosis (identification) by enhancing the effectiveness and intensity of therapeutic agents by preventing metastasis, and tumor invasion, and blocking the angiogenesis pathway. It is also concluded in the trial phase that it improves the therapeutic index even in patients with higher stages. It is utilized in emergencies 36. When all the benefits are considered, it is the least hazardous to non-target cells. This lessens the likelihood of unpleasant side effects like hair loss, anemia, nausea, cardiotoxicity, and many more 66. Tumors often have a high level of intricacy and heterogeneity. Because of heterogeneity, tumor development differs from person to person, and amazingly, even when the same individual has many tumors. As a result of this capacity, it does not react to therapy well and develops resistance to it 67. When compared to conventional chemotherapeutic medicines, nanotheranostics, which have both diagnostic and therapeutic properties, have a considerable deal of success. Its advantage can be studied in detail under the given sub-categories.

### Diagnostic and therapeutics

The specific marker for detecting HCC is AFP. However, it stimulates the growth of new cells, which advances the tumor. A nanotheranostics is created when an aptamer (NP) binds to AFP, reducing AFP's capacity to cause cell division 68. It gets picked up by SELEX (Systematic Evolution of Ligands by Exponential Enrichment) 69. The complex was found to be efficient, time-saving, and high-resolution. Magnetic NP with drugs such as Doxorubicin hydrochloride (anticancer) provides additional advantages such as minimal toxicity, a tiny, homogeneous geometrical size, good water stability, and distinctive MRI characteristics 70.

### Enhancing therapeutics

Due to multi-drug resistance (MDR), doxorubicin does not function as an anti-tumor agent alone. Overexpression of the ATP-binding cassette causes MDR 71. Reducing the function of ABC as a result of interaction with nanoparticles lowers the likelihood that a medication would cause MDR, improving therapeutic outcomes. Another illustration is the targeted delivery of Beclin 1 siRNA using calcium phosphate nanoparticles (NP) to boost the effectiveness of the therapeutic drug 72.

### Diagnostic and targeted-imaging

An activatable aptamer-based fluorescence probe (AAFP) is used to detect the presence of HCC. When a tumor is present, it produces a lot of light rather than emitting little fluorescence when one is absent. It is an effective method for spotting HCC early. It eliminates the requirement to maintain the optimum structure and additional moiety as fluorescent to get the desired result 73. Streptavidin is conjugated with carboxyl-modified FITC-doped silica nanoparticles, which results in exceptionally high specificity and decent sensitivity. No toxic effect of this complex was observed 74.

### Gene editing

CRISPR is an emerging element in both gene therapy as well as diagnosis. Sorafenib, a medication that has been clinically indicated as a multi-kinase inhibitor to treat HCC, was more effective in treating HCC cell lines when ERK2 kinases were inhibited by CRISPR/Cas9. In order to defend against viral DNA invasion, the CRISPR/Cas9 system was initially found in bacteria and archaea 75. Gene editing uses various vectors such as adenoviral vector, viral vector, lentiviral vaccine, RNA viral vector, exosomes and many more 76.

## Administration of interventional nanotheranostics

NPs are administered orally, through intravenous, pulmonary, transdermal, or intraarterial routes based on the type of cancer and part of the body affected by cancer. Among these, all routes of administration oral administration are the widely accepted route for the treatment of various cancers especially, in HCC. However, it is the most difficult path also. In the case of HCC, drugs can be administered through the oral and intraarterial routes of administration.

NPs that are mostly given by oral route has an advantage like localized drug delivery at the tumor lesion site, and as it is a non-invasive method, they can be easily administered. Apart from this protein and biological products can also be given in form of oral NPs formulations which can improve their bioavailability as compared to conventional but oral NPs formulation also faces challenges like first-pass metabolism, intestinal mucosal uptake, and microenvironment of tumor site, For the systemic delivery of NPs they are given by intravenous route but they also have disadvantages like first pass metabolism, and systemic toxicity and similarly other routes have also their pros and cons 77.

Oral administration of NPs is the most preferable mode in HCC. Research shows that Oral formulation of cargo-loaded NPs is more efficient and safer to treat targeted HCC than other routes. Other advantages of the oral formulation of NPs have enhanced bioavailability, increased drug solubility and stability, protection against pH, enzyme degradations, and other factors like patient compliance. A study on the efficiency and safety of apo transferrin(apodoxonano) and lactoferrin(lactodoxonano) NPs loaded with doxorubicin revealed improved bioavailability, safety, and efficacy when they are administered orally 78,79.

A category of therapy known as intra-arterial interventions for ineradicable HCC uses catheters to deliver medicated and /or embolic chemicals intra-arterially (via arteries) to specific tumor lesions. Intraarterial therapies include Selective internal radioembolization therapy (SIRT), transarterial chemoembolization (TACE), drug-eluting beads-trans arterial chemoembolization (DEB-TACE), bland embolization (TAE), and hepatic artery infusion 80,81. A study on intra-arterial administration of doxorubicin-loaded NPs revealed an enhanced overall life span but it also harms the respiratory tract [82, p.].

## Different nanoparticles used in nanotheranostics

NPs can be widely categorized into several groups based on their shapes, size, and chemical characteristics. Some of the different organic NPs, inorganic NPs, and hybrid NPs are used in cancer treatment. Organic NPs include liposome-based NPs, polymer-based NPs, and dendrimers whereas inorganic NPs include gold NPs, carbon-based NPs, silica NPs, magnetic NPs, and quantum dots. Hybrid NPs (least used) include lipid-polymer hybrid NPs, organic-inorganic hybrid NPs, and cell membrane-coated NPs 83,84. However, the NPs that are mostly used in the diagnosis and treatment of cancer are carbon-based, Lipid NPs, metallic NPs, silica NPs, quantum dots, nanoshells, dendrimers, and superparamagnetic FeO NPs 43. Additionally, certain phytochemicals that are utilized as nanocarriers have specialized actions including changing cellular composition and enhancing the effectiveness of anticancer medications 85,86.

### Carbon-based nanoparticles

Carbon-based NPs have been used to diagnose malignancy or administer medications to specific areas of the body. In carbon-based NPs, carbon nanotubes (CNT), fullerenes (FUL), graphene (GRP) and its derivatives, carbon-based quantum dots, nanodiamonds, and graphene oxides are taken into account as they are used as drug carriers, also in diagnosis and photodynamic therapy in cancer nanotheranostics. CNT has a hollow cylindrical, elongated shape with a diameter of 1-2 nm and distinct physiochemical and biological features which allows their utility in diagnosis and targeted drug delivery in HCC. CNT can be rolled into a single sheet, double sheets, and multi sheets. So that they are known as single-walled carbon nanotubes (SWCNTs), double-walled carbon nanotubes (DWCNTs), and multi-walled carbon nanotubes (MWCNTs) respectively. They also have a large capacity for anticancer drugs loading to their cylindrical shapes such as doxorubicin, methotrexate siRNA and paclitaxel can be delivered by CNT. Additionally, they are used in thermal ablation therapies because of their heat-generation ability when subjected to infrared radiation 87. Among the variety of NPs, CNT is the most suitable for biosensor applications due to its great mechanical strength, superior electrical conduction, optical and thermal properties, and capability to function as an effective signal transducer. As a result, they are used as a diagnostic tool to measure or quantify specific biomarkers in various tumors. Although CNT is still in its infancy because of contradictory findings regarding its toxic effects which may be because of variations in synthesis and other parameters that can affect drug delivery. Moreover, in *in-vivo* models, they show tissue build-up brought on by repetitive exposure to CNT due to their delayed disruptive kinetics. Thus, CNT is still a hot topic for researchers to explore its advantages and utilization in diagnosis and treatment 88-90.

Apart from CNTs, fullerenes and graphene are getting attention for their use in cancer treatment. Fullerenes are the pure form of carbons and are spherical. Fullerenes are varying in size; the largest form of fullerene contains 1500 atoms of carbon and the smallest one contains 20 carbon atoms. Generally, the C60 form of fullerene is widely acceptable and stable. With their considerable anticancer activity and great biocompatibility, adequately engineered fullerene NPs have a wide range of potential applications in anticancer therapy, especially in photodynamic therapy (PDT). fullerenes derivatives also have activity like promotion of the immune system, suppression of tumor angiogenesis, prevention of multiple drug resistance, and most important antioxidant activity. However, their utility in the medical field is limited due to their highly hydrophobic nature and propensity to agglomerate in aqueous solvents. This can be resolved by the derivatization technique in which polar groups are added to fullerenes 90-92. Graphene and its derivatives also possess numerous exceptional and distinct physical and chemical features; hence they are frequently developed as revolutionary molecules to incorporate into various therapies such as phototherapy (photothermal and photodynamic therapy), chemotherapies, and imaging tools for tumor treatment 93,94. In conclusion, carbon-based various NPs in cancer therapy are emerging fields right away.

### Lipid-based nanoparticles

One of the most popular NPs for HCC nanotheranostics is lipid-based nanoparticles such as liposomes, solid lipid NPs (SLN), and nanostructured lipid carriers (NLC). Among these, liposomes are the most widely used in cancer therapy. Liposomes are also known as vesicles or simple lipids and are typically between 50 to 200 nm in size and spherical shaped. Phospholipids and cholesterols are the basic need to create liposomes 95,96. Liposomes comprise a lipophilic bilayer and the hydrophilic core part which enables the encapsulation of hydrophobic and hydrophilic drugs into liposomes 97. Generally, hydrophilic material encapsulates in the core part whereas, lipophilic material is incorporated into the lipid bilayer. Doxorubicin, sorafenib, and siRNA are often encapsulated in various types of liposomes. The main purpose of encapsulation is to protect the active drug from degradation by the immune system 96,98. Numerous biological applications, particularly in the field of drug administration, have been made possible by the distinctive qualities of liposomes, such as biocompatibility, biodegradability, amphiphilicity, less toxic effects, non-ionicity, prolonged drug release, and site-specific action 96,99. Liposomes are mainly used as drug carriers because of their superior carrier qualities and mobility. The clinical data of PEGylated liposomes loaded with doxorubicin revealed increased efficacy in the treatment of HCC because of its ability to carry the drug safely to the HCC cells. They are also assisting with imaging diagnoses and therapies for cancers. Additionally, they are also used in combination therapies 88. Liposomes' surface has frequently been modified to increase their circulation and distribution in the blood which ultimately improves the efficacy of the anticancer drug 100. For example, HCC cells expressed specific receptors like the asialoglycoprotein receptor (ASGPR) which is selective for glycoproteins, particularly D-galactose or galactosamine. Thus, to target paclitaxel anticancer drug to HCC cells, scientists created unique ASGPR-targeting poly (polyethylene glycol paclitaxel) (PTX) nanoliposomes that contain PTX and active ligands which directly strike HCC cells. As a result, it improved drug efficacy and decreases the toxic effect of the drug on a cell other than HCC cells. Apart from surface engineering, attachment of targeting agents and polymer coating also improve targeted drug delivery. For instance, SP94‑targeted PEGylated liposomal DOX (SP94‑LD) which contains SP94 peptide onto polyethylene glycolate polymer coated (PEGylated) liposomes loaded with doxorubicin, shows tissue accumulation near cancerous cells by EPR effect and active targeting effect 88,96. However, liposome interaction with cancerous cells is affected by liposome composition, diameter, charges present on surfaces, etc. 101. Currently, there are many liposomes' products available on the market that are approved to treat different diseases, and there are still researches ongoing to making stable liposomes because physical and chemical stabilities of liposomes formulation is the major obstacle. So, it's necessary to develop stable and efficient liposomes 102.

### Metallic nanoparticles

Metallic NPs have a potent and advantageous impact on cancer therapy as they have the potential to be used as multipurpose agents, and investigation raising continuously on metal NPs. Examples of metallic NPs include Gold NPs (AuNPs), silver, magnesium, iron, copper, calcium, zinc, and many more. Among them, Gold NPs are the leading nanoparticles right now 103,104. AuNPs are available in different shapes (nanoshell, nanorod, nanocage, hollow nanosphere, and many others) and sizes of about 25-200 nm. Owning various physical, chemical, optical, electrical, and biological properties, AuNPs are applicable in a wide variety of scientific fields including medicine. Functionalized AuNPs i.e., attachment of different molecules like anticancer drugs, antibodies, enzymes, fluorescent dyes, etc on the surface of AuNPs directly reflect in their conjugation, cellular uptake, cytotoxicity, and interaction with cancerous cells. research studies on AuNPs show that their toxicity can be correlated to size, surface functionality, and charges present on the surface 105,106. AuNPs are good contrast agents because of the great absorbance of X-rays in the imaging diagnosis of tumors. AuNPs also has potential application as drug carrier and in gene therapy. Additionally used in phototherapies (including PTT and PDT), and radiosensitizer 38,107,108. The use of gold nanoparticles as cancer nanotheranostics must overcome several obstacles, including biodistribution, pharmacokinetics, and potential toxicity 109. As we earlier mentioned, Apart from AuNPs many other metallic NPs are also utilized for cancer treatment. The use of NPs as interventional nanotheranostics tools has increased over the past few years, and further research is still required to develop more potent and efficient treatments for hepatocellular carcinoma.

## Drugs under clinical trials

**Figure 2:**
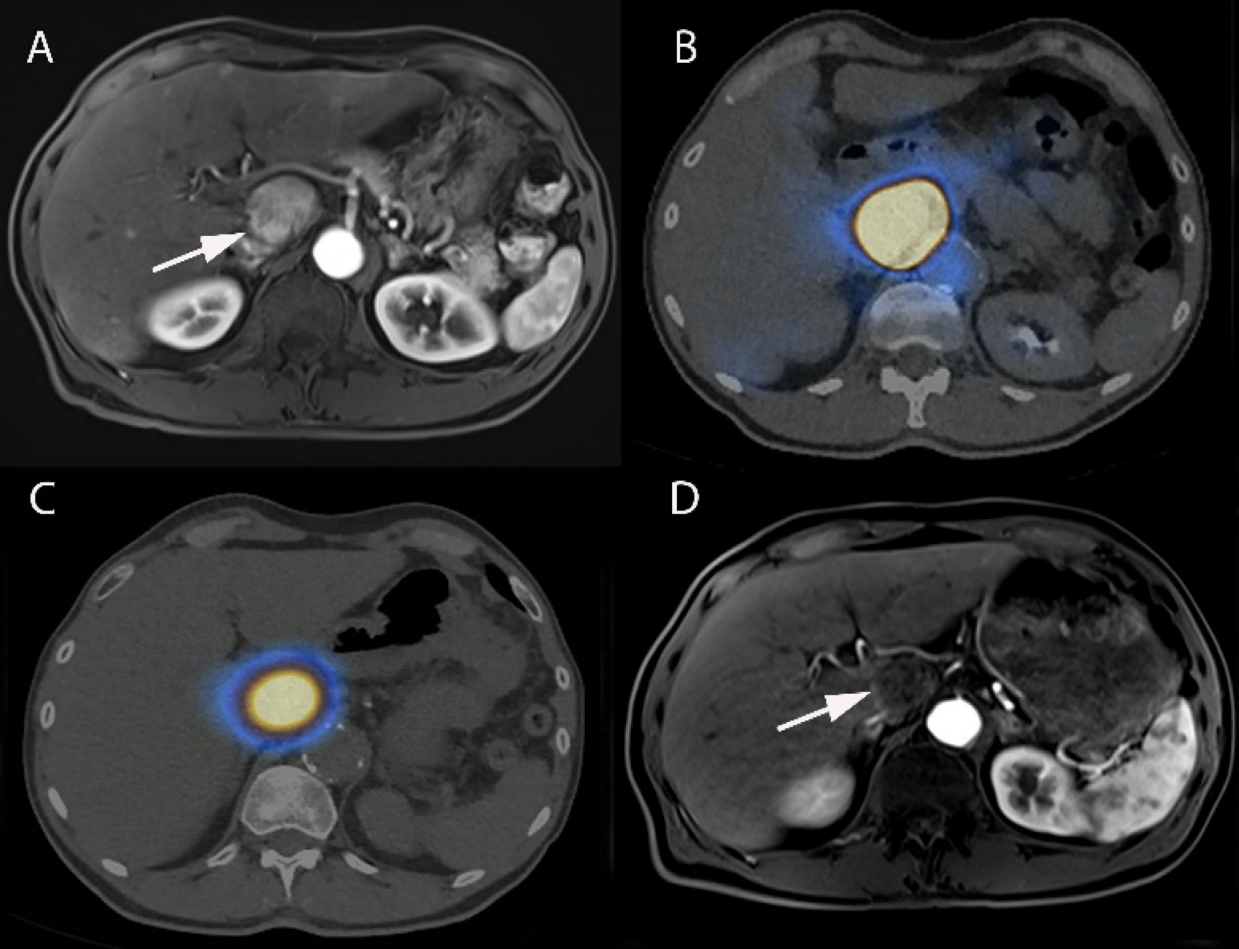
** Interventional nanotheranostics treatment in a 63-year-old-male HCC patient. (A)** It demonstrates the growing tumor in the caudate lobe near the HCC (arrowed part). **(B)** The pre-treatment with 99mTc-MAA describes the super-selective accumulation in caudate lobe. **(C)** Because of the pre-treatment with 99mTc-MAA, the targeted delivery of 90Y-microsphere (therapeutic agent) shows excellent tumor targeting. **(D)** The image shows a complete disappearance of growing tumor after 3-month therapy with SIRT because of the targeted delivery. (Adopted under CC by 4 licences from 112).

A clinical trial is required to prove the effectiveness of the drug and its combinations. Clinical trials, with the help of advance technology and living volunteers, such as animals named rodent and smaller animals, and human volunteer, both healthy as well as patient, ensures the better understanding of the moiety and help to reduce the gap between theoretical assumption and actual effect. It describes the multi-target effect of molecule 110. Refer Figure 2 for a case study illustrating the effect of nanotheranostics in HCC patient.

Before the actual treatment, diagnosis of angiography is crucial. The treatment stimulant, technetium-99m-macroaggregated albumin (^99m^Tc-MAA), is administered in the body which is detected by single-photon emission CT (SPECT) to evaluate the dose required for the treatment of tumor 111. This step is crucial as it leads to personalized delivery of drug to the targeted site 112.

Along with the being a helping hand in targeted delivery, it has its own therapeutic efficacy. Some of them are described in the given Table 1.

HCC is typically diagnosed at an intermediate or late stage. An effective treatment is required to prevent further loss of liver functions in these stages. According to the BCLC classification of HCC in an intermediate stage, an asymptomatic, multinodular, noninvasive liver is observed. To treat the intermediate stage of HCC, transarterial chemoembolism (TACE) is preferred as a standard treatment. TACE is administered via percutaneous injection. First of all, a catheter is inserted in the lumen of a blood vessel, which is used to carry the cytotoxic drug only to the cancerous cells of the liver. The name “chemoembolism” itself suggests that chemotherapeutic agents with embolizing agents are involved in this treatment. Chemotherapeutic agents destroy the cancerous cells, while embolizing agents stop or block the blood vessel temporarily to perform the TACE procedure. Cytotoxic drugs that are given to treat tumour cells are emulsified with lipoidal (contrast medium), followed by embolization 113-115.

TACE can be performed by two distinct methods: conventional TACE or by using drug-eluting beads with TACE (DEB-TACE). The former method is normal and includes anticancer drug delivery followed by embolization. whereas the second method includes drug-eluting beads (DEB), which slowly release the anticancer drug to enhance the intensity and drug exposure at the cancerous site. Hence, it modifies the pharmacokinetic profile of treatment, which is not the case with conventional TACE. Clinical research data on DEB loaded with doxorubicin shows less systemic and liver toxicity 116-118.

The thermosensitive liposomal formulation of doxorubicin is commercialized under the tradename ThermoDox. Doxorubicin is the first agent in the thermal-sensitive category of liposome formulations that are tried in clinical studies. In clinical trials, ThermoDox is used in combination with radiofrequency ablation (RFA) to potentially evaluate the effect of formulation at the HCC tumor site. RFA is used in combination because it provides mild thermal energy and can cause hyperthermia which ultimately enhances ThermoDox effectiveness. The OPTIMA trial is conducted first to evaluate ThermoDox efficacy when used in conjunction with RFA as compared to the use of RFA therapy alone.

**Figure 3:**
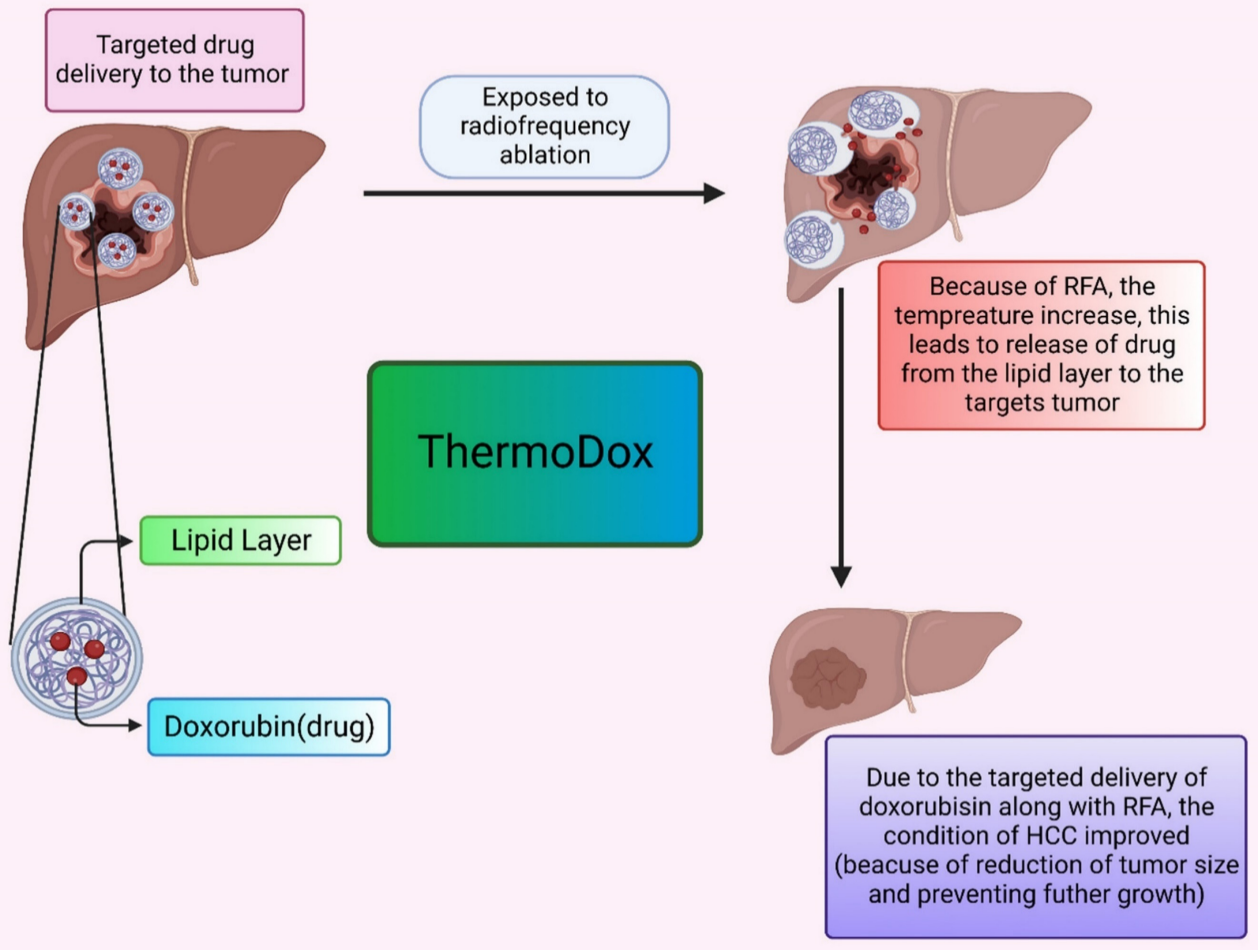
**The delivery of the drug by ThermoDox.** It describes the effect of elevated temperature leads to release of drug from the lipoidal moiety. This effectively decreases the tumor.

ThermoDox is a liposomal formulation with a doxorubicin payload as an active anticancer drug. The mechanism behind the action of this thermosensitive liposomal formulation is, when they are subjected to heat, they release drugs at the tumor site by the opening of pores of the lipid bilayer or enhancing lipid bilayer permeation. RFA provides heat that causes a rise in body temperature up to 42 Celsius and an increase in body tissue temperature of approximately 39 to 42 Celsius is known as hyperthermia. blood flow and oxygenation in body tissue are high in hyperthermia which is beneficial in drug delivery. For the pictorial presentation, refer **Figure *3***. Moreover, ThermoDox with RFA therapy produces a high drug concentration of doxorubicin as compared to free doxorubicin near the ablation zone due to hyperthermia 119,120,121.

The formulation of ThermoDox was made of Lysphospholipids, which ensures the phase transition temperature of liposomes is lowered to around 40 °C. As a result, when heated, they can be regulated to change their structural makeup and release the drug payload through openings in the liposome (Dox). The ThermoDox formulation also has a short half-life so it required immediate thermal ablation therapy after administration of formulation to achieve its maximum effect The slow drug release of this formulation was a major challenge and as it releases the drugs only above the transition temperature of liposome, it releases the maximum 70% of drugs from the formulation. These factors contributed to the failure of clinical trials of ThermoDox formulation 122,123,124,125.

There is a vast area for research and development in such a miraculous moiety. This review clamor to more trials and studies on interventional nanotheranostics concerning HCC to overcome the drawbacks of current healthcare systems.

## Current hurdles in using nanotheranostics and future aspects

Nanotechnology has grown immensely in the last few decades. It is applicable in almost all fields including engineering and medical also. NPs are used to diagnose and treat various diseases especially, in cancer treatment nanotheranostics have a great impact on cancer treatment than conventional drugs. With the advancement in nanotechnology, we can potentially improve cancer treatment however, right now we are facing many challenges in development and its potential use in imaging and treatment.

Some of the major hurdles in the development of NPs are particle sizes and shapes, characteristics of NPs, components, and analysis of NPs formulation, regulatory and pharmacological challenges as well as safety and efficacy requirements for approval of NPs 32,38. Nanomedicine or NPs need to be thoroughly studied in its investigation stages because every minor change can significantly affect NP's safety and efficacy. Moreover, particle size directly affects its biodistribution, drug PK and effectiveness of NPs. For example, Smaller NPs (20-30 nm) rapidly undergo renal clearance while particle size 200 nm or larger than that is actively taken up by organs like the liver and spleen, particularly particle sizes ranging between 150-300 nm. Particle size also varies depending on the route of administration for instance particle size should be small in intravenous than in oral formulation 39,40,42. Half-life also had an impact on the treatment of cancer. NPs enter tumor cells via leaky blood vessels so if NPs have a longer half-life, they remain in blood circulation for a longer period then there are more chances of entering of NPs into cancerous cells and enhanced treatment outcomes. Additionally, with the help of PEG rapid clearance can be reduced. Hence, to achieve a long half-life PEG is attached to the NP's surface but excessive use might be disadvantageous 41. pharmacological effects prediction is the presently major obstacle in the development of NPs since there are currently no reliable *in vivo* models to forecast the wide range of behaviours of the various types of nanoparticles being studied, the creation of nanoparticles with desirable features has to rely on empirical data and substantial preclinical animal testing. Similarly in the context of analysis and characterization, due to the complex structure of nanomedicine, it required more sophisticated testing to understand different functional groups and their nature for example, to what extent active drug-loaded NPs can bind to specific targeted receptors and cells and what modification needs to be done for more effectiveness 32. A nonspecific build-up of nanoparticles could harm healthy tissue in cancer treatment that uses external sources of energy like ionizing radiation or infrared radiation. This threat can be reduced by using image-based management in the *in vivo* model, in which NPs dispersion has to be assessed. So, for these types of energy-based treatment, the distribution of medications supplied by NPs can be examined simultaneously using imaging techniques that have not yet been fully explored by researchers. Hence, to observe the activation of NPs straightforward imaging technology need to be explored 43. Apart from these active targeting of NPs also hurdles in the treatment of cancers. NPs are actively directed to the specific cancerous cells by adding some targeting moieties such as antibodies, ligands, transferrin, etc. to the surface of NPs. These will help to interact with molecules or receptors present on the surface of the targeted cell. Hence, the ratio of interacting moieties and targeted surface molecules should be studied to efficiently cross the barrier and cellular uptake and these make active targeting more difficult. Moreover, the target selection process is personalized because disease molecules and biomarkers vary in every patient and their disease condition 44. In the present scenario, the manufacturing of NPs is another big obstacle. it's important to note that nanoparticles are not just simple combinations and additions of different parts. Instead, they are comprehensive compositions with a complex structure. Throughout the formulation process and until the final product, the structural, physical, and chemical characteristics of integrated NPs must be maintained because numerous factors can affect manufacturing, for example, nanomaterial storage temperature, pH, humidity, stability, sterile condition, and other processing methods including proper solvent selection and method of preparation. Therefore, full care should be taken to make efficient and affordable NPs products 38,45,49. Despite all these hurdles, NPs research is continuously raising especially in tumor treatment and if scientists will overcome these problems, NPs therapy can be used as a standard and promising to decrease the mortality rate of cancer.

## Conclusion

Nanotheranostics is the emerging field that ensures the safe delivery and management of the disease. It works on a molecular as well as the cellular level to enhance the effect of the drug and decrease the level of extracellular toxicity. There is various particle that is used to serve this purpose, gold NP, liposome-based NP, and many more. This particle has a variety of uses, including targeted delivery, medical diagnostics, lowering toxicity, and many more. There are still many of opportunities for development and study to gain a deeper comprehension of ideas that might provide great achievement. Nanotheranostics may be used to successfully predict the future of many fatal diseases. It will provide the secure administration of many powerful medications, whose action on healthy cells makes them dangerous at the moment. The effective medication delivery method can successfully overcome this obstacle.

## Figures and Tables

**Table 1:** Clinical studies on the treatment of HCC with interventional nanotheranostics

Study Ids	Status	Phase	Study design	Study Start	Study Completion	NCT Number
SH3137	Not yet recruiting	Phase 2	Interventional Single group assignment Primary Purpose: treatment	December 2021	December 2024	NCT05161143
JNZY20181245	Recruiting	Phase 1	Interventional Randomized Parallel Assignment Primary Purpose: treatment	September 1, 2018	December 31, 2023	NCT03652467
IIT-0027	Not yet recruiting	Phase 2	Interventional Single Group Assignment Primary Purpose: treatment	October 1, 2022	October 1, 2028	NCT05451043
OTX-2002	Recruiting	Phase 1 Phase 2	Interventional Non-Randomized Parallel Assignment Primary Purpose: treatment	August 19, 2022	December 2028	NCT05497453
CA209-678	Active, not recruiting	Phase 2	Interventional Single Group Assignment Primary Purpose: treatment	December 20, 2016	December 31, 2022	NCT03033446
UW 18-541	Active, not recruiting	Phase 2	Interventional Single Group Assignment Primary Purpose: treatment	March 1, 2019	September 5, 2022	NCT03817736
2020-212	Recruiting	Not available	Observational Observational Model: Cohort Time Perspective: Prospective	November 19, 2020	December 31, 2022	NCT04682847

## Abbreviations

AAFP: aptamer-based fluorescence probe
ABC: antibody biopolymer conjugate
AFP: alpha-fetoprotein
ASGPR: asialoglycoprotein receptor
BCLC: barcelona clinic liver cancer
CNT: carbon nanotubes
CRISPR: clustered regularly interspaced short palindromic repeats
CT: computed tomography
CYP: cytochrome
DEB-TACE: drug-eluting beads-trans arterial chemoembolization
DNA: deoxyribonucleic acid
DWCNTs: double-walled carbon nanotubes
ERK2: extracellular signal-regulating kinases
FeO: ferric oxide
FLL: focal liver lesion
FUL: fullerenes
GNPs: gold nanoparticles
GRP: graphene
HBV: hepatitis b virus
HCC: hepatocellular carcinoma
HCV: hepatitis c virus
MDR: multi-drug resistance
MRI: magnetic resonance imaging
MWCNTs: multi-walled carbon nanotubes
NAFLD: non-alcoholic fatty liver disease
NLC: nanostructured lipid carriers
NPs: nanoparticles
PDT: photodynamic therapy
PEG: polyethylene glycol
PTX: polyethylene glycol paclitaxel
RFA: radiofrequency ablation
RNA: ribonucleic acid
SELEX: systematic evaluation of ligands by exponential enrichment
siRNA: small interfering rna
SIRT: selective internal radioembolization therapy
SLN: solid lipid nanoparticles
SWCNTs: single-walled carbon nanotubes
TACE: transarterial chemoembolization
TAE: bland embolization
99mTc-MAA: technetium-99m-macroaggregated albumin
TERT: telomerase reverse transcriptase
VEGF-R: vascular endothelial growth factor receptor
